# Case of Death from Fat Embolism after Bone-Setting

**Published:** 1884-03

**Authors:** Arthur W. Prichard

**Affiliations:** Surgeon to the Bristol Royal Infirmary


### CASE OF DEATH FROM FAT EMBOLISM AFTER BONE-SETTING. By ARTHUR W. PRICHARD, Surgeon to the Bristol Royal Infirmary.

I THINK the following case may be of interest and warning in showing a great danger that may attend any forcible movement of an ankylosed joint, especially if that joint has suffered from chronic inflammation and has for the most part been treated by rest. The unfortunate ending of the case was death in a few hours, and this result was directly due to bone-setting, by which I mean forcibly moving under chloroform a stiff joint. Not more force was used in this instance than was necessary, I think not as much as is usual; yet an injury was inflicted on the articular ends of bones owing to their being softened and wasted by being kept in splints too long, and this injury caused the death of the patient.

*Prima facie* the case looks like one of opium poisoning, and until the microscopic examination of the lungs and kidneys, all who saw the patient, I believe, attributed death to this cause. The credit of suggesting fatty embolism as the cause of death is due to Mr. Greig Smith, and on examination of the lungs and kidneys it was demonstrated that his suggestion was correct, both these organs being full of emboli of particles of fat.

The case is as follows:—C. P., aged 18, a fat girl, was admitted under me at the Bristol Royal Infirmary a year ago suffering from chronic synovitis of the knee. She had had pain and swelling in it for three years, and had been lying in bed nearly all that time. On admission the whole limb was thickened and stiff. She could not bear the slightest attempt at bending the knee, and any movement of the limb gave her pain in the ankle, knee and hip. There was no albumen in the urine. I examined her under chloroform and found the hip quite free, but the knee joint stiffened and in fairly good position. I did not attempt to move it, but put her on a McIntyre splint and tried the effect of Scott's dressing and counter-irritants. After the examination she had increased pain, and for some time had a draught every night of 40 drops of liq. morphiæ hydrochloratis, which on some occasions was doubled without producing any bad effect. After three months, as there was no marked improvement, she was sent for a while into the country.

She was re-admitted in the June of the present year, and was much in the same state as when she went out. I again tried various plans of treatment, such as slight manipulation, followed by strapping, elevation, Scott's dressing and iodine, but no improvement ensued. As everything tried had been of no avail, after consultation with my colleagues, I agreed that movement of the joint under chloroform might possibly do something for her.

Accordingly, on Sept. 14, at 1.30 p.m. she was put under the influence of chloroform and ether, and I examined the limb. There was tolerably firm ankylosis of the knee and stiffness of the ankle. I took hold of the femur with my left hand, and the tibia about its middle with my right, and tried to bend the knee, but as the adhesions did not give way after using some force, I put my left arm under the popliteal space and slowly flexed the joint over it. I felt some bands in the joint give way with that peculiarly unpleasant sensation that attends the breaking down of adhesions in or around a joint, and then after a little more flexion a firmer structure gave way, which enabled the lower part of the leg to move freely. I had not exercised much force, but the free lateral mobility that followed made me afraid that I had separated the epiphysis of the tibia—which turned out to be the case. The limb was bandaged from the toes, and the patient carried back to bed, the leg being kept still by sand bags. The girl had been under chloroform a very few minutes, and came round in the usual time afterwards. There was no vomiting.

In the afternoon she complained of severe pain in the knee and leg, and at five o'clock was seen by the House Surgeon and ordered 40 drops of liq. morphiæ.

At 8.30 the patient was still complaining; she had had no relief from the morphia and was very restless. The limb was examined and found to be much congested, the foot being cold. The posterior tibial artery could be felt pulsating. The bandage was removed, hot bottles placed in the bed, and 20 drops more of liq. morphiæ given.

Two hours afterwards she was still in severe pain; there had been no sleep; the foot was blue and cold, the rest of the body being normal in colour. The pupils were not contracted. 20 drops more morphia were ordered.

At 1 a.m., two and a half hours after the last dose of morphia, she was sitting up in bed complaining bitterly of the pain. She had had no relief from it at all since the operation, and 20 drops more were given. A quarter of an hour afterwards she went to sleep, and the night nurse, who did not leave the ward, noticed her to be sleeping quietly and not making any noise in breathing.

At 2 o'clock she was visited and seen to be lying quietly asleep.

At 3 the pillows were moved and the patient turned over in bed, but did not wake up; there was no blueness of the face, and she slept quietly till about 5. At 5 her sleep was observed to be heavy, and the house surgeon saw her at once. There was then considerable cyanosis of the face and of the affected limb; the pupils were much contracted, and the conjunctivæ insensible. The patient could not be roused. The breathing was rapid and deep, and moist sounds were to be heard all over the chest; a little frothy mucus was coming from the mouth, and the pulse was small, quick, and regular.

She was at once transferred to a private ward and the stomach pump was used, the stomach being washed freely out. Strong coffee was administered and forced movement employed, the patient being rolled about in bed. Cold affusion and flagellation were tried, as well as galvanism and artificial respiration. This treatment was continued for some hours, and twice a hypodermic injection of one-thirtieth of a grain of atropine given. The rapidity of the respiration kept up, her breathing being shallow, but as many as 30 in the minute. There were mucous râles all over the chest. The urine was drawn off and found to contain about one-fourth albumen, and the patient gradually sank and died about one p.m., nearly twenty-four hours after the operation. Just before death there was considerable twitching of the arms, and the pupils dilated widely, but she never regained consciousness.

The post-mortem examination took place next day, and to the naked eye no sufficient disease in the internal organs was found to account for death. The lungs were hyperæmic, and it was fancied that the blood which escaped on their section was greasy to the touch, and the kidneys were congested, the capsule being somewhat adherent, the cortical portion diminished, and there was a cyst in one. The knee-joint on being laid open was seen to be full of thin blood; there was a recently broken adhesion between the internal condyle and the inner tuberosity of the tibia, and the cartilage was gone from a part of the internal condyle, leaving the bone exposed and rough for three-fourths of a square inch. The compact layer of the whole of the lower end of the femur was exceedingly thin, and so soft as to take the imprint of the nail easily. It was indented at one part, and on section with the knife the cancellated structure in the vicinity of the dent on the internal condyle was filled with fat and dark blood, the outer condyle or section presenting cancellous structure filled with yellowish marrow. The tuberosities of the tibia were also soft, and the tibia was fractured through its upper epiphysis. The popliteal and femoral arteries and veins, the common iliac vein and the vena cava were examined and found healthy.

Fatty embolism was now suggested as being the cause of death, and the lungs and kidneys were subsequently examined by Dr. Fenton Evans and Mr. Greig Smith, and it was found that both organs presented in every part well marked blocking of the capillaries by fat globules.

We therefore concluded that fat embolism combined with morphia was the cause of this patient's death. Morphia, in the amount that was given, would probably not have caused it alone. She had five-sixths of a grain in divided doses between five in the afternoon and one o'clock next morning, and until the last twenty drops were given she had had no sleep; and this was the same patient who had on previous occasions taken two-thirds gr. in two doses in a much shorter time, without bad effect. Further, the greater the pain the more opium a patient can bear with impunity, and the pain that this girl suffered after the operation was apparently far more than ever she had had before. Also, there was one symptom before death that showed that death was not due to morphia poisoning alone, namely—the rapidity of breathing that was kept up till she sank. This made us look for some other cause, and I think that there is no doubt but that fatty embolism was the chief cause of death.

It is easy to see how this embolism might arise. The veins from the cancellous part of the articular ends of a long bone emerge by numerous large apertures in the compact layer, and having support from bone have very thin walls, and are held open if torn across. Some of these vessels in this case, on account of the weakened bone being unable to resist the pressure applied during manipulation were broken, and a communication was opened up between the cancelli, filled with fluid marrow, and the veins. Blood flowed freely into the cancellated structure, as was proved by the ecchymosed condition the condyle was in when cut open, and fluid marrow probably escaped by the veins and was circulated generally through the system.

If such be the explanation, it is remarkable that the accident does not more frequently occur in ordinary fractures, and I think it is likely that it does happen more often than the few records of fatty embolism would lead us to believe; death ensuing soon after a fracture is put down to shock perhaps too often. Further, is it not possible that the condition of fatty embolism may occur, yet the patient recover? In such a case the symptoms would be masked, and the danger of the constitutional disturbance that follows any serious injury to bones increased. Fatty embolism in such a case, causing perhaps hyperæmia and œdema of the lungs, would be rendered much more dangerous to life by the administration of opium to the patient, for this would increase the difficulty that they had to contend with by retarding the respiratory movements, and yet the condition a patient is in immediately after a fracture is more commonly treated by that drug than by any other.

Should a case of fatty embolism be diagnosed, I venture to suggest that ether, either inhaled or swallowed, would be the rational treatment; it would be beneficial as a stimulant, perhaps dissolve or more finely divide the fat in the vessels, and help its elimination by the kidneys.

In conclusion, fracture, or, if I may so call it, laceration of soft bone is not an uncommon accident to those who promiscuously work over-rested joints about, and I think that the occurrence of fatty embolism is a possibility to be remembered; and if more attention were called to this cause of death, in fractures, osteomyelitis, and other diseases and injuries of the bones, more cases of it might be recorded.

